# Closed-Loop Automated Oxygen Control in Preterm Infants Receiving Non-Invasive Respiratory Support

**DOI:** 10.3390/children12111528

**Published:** 2025-11-11

**Authors:** Ourania Kaltsogianni, Theodore Dassios, Anne Greenough

**Affiliations:** 1 Women and Children’s Health, School of Life Course and Population Sciences, Faculty of Life Sciences and Medicine, King’s College London, London SE5 9RJ, UK; ourania.kaltsogianni@nhs.net (O.K.); theodore.dassios@kcl.ac.uk (T.D.); 2 Neonatal Intensive Care Centre, King’s College Hospital NHS Foundation Trust, London SE5 9RS, UK

**Keywords:** closed-loop automated oxygen control, neonate, non-invasive ventilation

## Abstract

**Highlights:**

**What are the main findings?**

• Closed-loop automated oxygen control (CLAC) systems improve the achievement of oxygen saturation targets in preterm infants on non-invasive ventilation.
• The evidence regarding prolonged use of CLAC is limited, and there are no reports on clinical outcomes.

**What are the implications of the main findings?**

• Future studies should explore the effect of the prolonged use of CLAC with different modes of non-invasive ventilation on oxygen saturation targeting and long-term outcomes.

**Abstract:**

**Background/Objectives:** Closed-loop automated oxygen control (CLAC) systems improve compliance with oxygen saturation targets and other outcomes in preterm ventilated infants. This narrative review aimed to explore the efficacy of CLAC systems in preterm infants receiving non-invasive respiratory support and identify areas that needed further research. **Methods:** A literature search was conducted using PubMed. The search terms were ‘closed loop’ or ‘automat*’, ‘oxygen’ and ‘neonat*’. **Results:** Sixteen studies were identified: twelve randomised crossover studies, three randomised controlled trials (RCTs) and a matched-cohort study. Nine studies included only infants receiving non-invasive respiratory support, and the remaining seven incorporated results from infants either on invasive or non-invasive ventilation. Overall, CLAC was associated with an increased percentage of time spent within the target oxygen saturation range and reduced time spent in extremes of oxygenation (SpO_2_ < 80% and SpO_2_ > 98%) when compared with manual oxygen control. CLAC was applied in infants receiving different modes of non-invasive respiratory support, including continuous positive airway pressure, high and low-flow nasal cannula oxygen. Some of the studies had limited power as they were prematurely stopped due to recruitment or equipment issues. Study periods were mostly less than or equal to 24 h. There were no data on longer-term clinical outcomes, including bronchopulmonary dysplasia, retinopathy of prematurity, necrotising enterocolitis and mortality. **Conclusions:** CLAC improves the achievement of oxygen saturation targets in preterm infants receiving non-invasive respiratory support. Future research is needed to explore the effect of CLAC on clinical outcomes in this population.

## 1. Introduction

Preterm infants frequently require supplemental oxygen, but, although its use can be life-saving, it can increase the risk of complications [1]. Hyperoxia increases oxidative stress [2] and increases the risks of bronchopulmonary dysplasia (BPD) and retinopathy of prematurity (ROP) [3,4]. Hypoxia increases morbidity and mortality [5,6].

Targeting oxygen therapy to maintain peripheral oxygen saturation levels (SpO_2_) within a predefined range can reduce the risk of complications. Compliance with achievement of oxygen saturation targets, however, has been shown to be rather poor and variable [7].

Closed-loop automated oxygen control (CLAC) systems may represent a solution to poor compliance and may, therefore, reduce complications. CLAC systems monitor oxygen saturation values in real time to calculate and make an adjustment to the inspired oxygen concentration (FiO_2_) without any human intervention [8]. Several algorithms exist to model and account for the relationship between SpO_2_ and FiO_2_ in infants with different severities of respiratory disease [9]. Studies have demonstrated that automated compared to manual oxygen control systems in preterm ventilated infants result in an improvement in the achievement of oxygen saturation targets, reduced time spent in hypoxia and hyperoxia and fewer manual adjustments to the inspired oxygen concentration [10,11,12,13,14,15,16,17,18,19,20,21,22,23,24]. In addition, CLAC has been associated with earlier weaning of the inspired oxygen [19,24]. In a randomised controlled trial (RCT) in preterm ventilated infants (n = 69), CLAC was associated with shorter durations of mechanical ventilation (MV) and supplemental oxygen and reductions in the incidence of BPD and the proportion of infants discharged on home oxygen [25].

Preterm infants are increasingly managed on non-invasive respiratory support with an aim to minimise lung injury and reduce respiratory morbidity [26]. Optimisation of supplemental oxygen treatment could further improve respiratory outcomes in this population. This narrative review aims to explore the efficacy of CLAC systems in preterm infants receiving non-invasive respiratory support and highlight areas for further research.

## 2. Methods

A literature search was conducted in PubMed for articles related to the use of CLAC in preterm infants. An advanced search was performed with a combination of search terms ((closed-loop) or (automat*) AND (oxygen) AND (neonat*). The reference list of relevant articles was also reviewed. We included published studies that compared closed-loop automated oxygen control to manual oxygen control in preterm infants (22 to 36 + 6 weeks of gestational age) on non-invasive respiratory support. Studies on CLAC that included only infants on invasive mechanical ventilation and studies comparing different automated oxygen controllers were excluded.

## 3. Results

Sixteen studies were identified that included preterm infants on non-invasive respiratory support: twelve randomised crossover studies, three RCTs and a matched-cohort study (Table 1). There were no studies involving term-born infants. A variety of algorithms, oxygen saturation targets, timing and durations of monitoring periods were used.

### 3.1. Current Evidence for the Use of Automated Oxygen Control in Preterm Infants on Non-Invasive Respiratory Support

#### 3.1.1. CLAC with Non-Invasive Respiratory Support

Three randomised crossover studies compared CLAC with manual oxygen control in preterm infants with respiratory distress syndrome (RDS) who were receiving continuous positive airway pressure (CPAP) or high-flow nasal cannula (HFNC) oxygen [17,22,28]. The studies demonstrated an improvement in the achievement of oxygen saturation targets [17,22,28], reduced frequency and duration of hypoxemic [22,28] and hyperoxemic episodes and fewer manual adjustments to the FiO_2_ with automated oxygen control [17,22,28].

In a two-centre, randomised crossover study, Schwarz and co-workers compared two versions of a CLAC algorithm, which had different response times to SpO_2_ changes (CLAC_fast_ and CLAC_slow_) with manual oxygen control. The revised, faster algorithm for CLAC, which allowed more frequent FiO_2_ adjustments, improved SpO_2_ target achievement (68% versus 58%; *p* < 0.001), reduced the percentage of time spent below the targeted range (14% versus 22%; *p* < 0.001) and the number of manual FiO_2_ adjustments compared with manual control (*p* = 0.08). Moreover, there was a trend towards less time spent below oxygen saturation targets (SpO_2_ < 90%) with CLAC_fast_ compared with CLAC_slow_ (22% versus 17%; *p* = 0.16), suggesting that faster responsiveness may be more effective [27].

More recent data support that the efficacy of CLAC in infants receiving non-invasive respiratory support may differ over time. A single-centre RCT on the prolonged use (28 days) of automated oxygen control in extremely preterm infants found an increase in the time spent below the target range (SpO_2 _< 88% and SpO_2 _< 90%) in the first days of life that gradually declined. The opposite trend was observed during manual oxygen control [29].

#### 3.1.2. High- or Low-Flow Nasal Cannula Oxygen

The beneficial effects of automated oxygen control on oxygen saturation targeting have also been observed in infants receiving high-flow nasal cannula (HFNC) oxygen treatment [24,30,31,32]. A RCT in extremely low birth weight infants on day eight to nine of life, showed that automated oxygen control over 12 h was associated with improved SpO_2_ target achievement (58% versus 33%; *p* < 0.01) and a lower time spent in hyperoxia (SpO_2_ > 95%; 26.5% versus 54.8%, *p* < 0.01) but an increase in the percentage of time spent with SpO_2_ < 85% (14% versus 11%; *p* = 0.05). The frequencies of prolonged hypoxic episodes and the incidence of more severe hypoxemia (SpO_2_: 70–75%) were reduced [30]. In another study, the mean FiO_2_ was higher during automated oxygen control (mean difference 0.019 (95% CI 0.006 to 0.03); *p* = 0.003), but there was no significant difference in the time spent in severe hyperoxia (SpO_2_ > 98%). This was attributed to staff training in how to avoid hypoxia and a potentially oversensitive response of the CLAC algorithm that led to overshoot and hyperoxic episodes [31]. A larger RCT that enrolled participants within 72 h of life and for the whole duration of HFNC treatment demonstrated a 26% increase in the percentage of time spent within oxygen saturation targets (*p* < 0.001) and reduced the time spent in extremes of SpO_2_ (<80% and >98%; *p* = 0.002 and <0.001, respectively) [32].

#### 3.1.3. CLAC with Mechanical Ventilation or Continuous Positive Airway Pressure (CPAP)

Many studies compared CLAC with manual oxygen control in preterm infants receiving either invasive mechanical ventilation (MV) or nasal CPAP. The majority of them have a randomised crossover design [12,20,23,27,33], and another two studies were parallel arm randomised controlled trials [34,35]. Two multi-centre randomised crossover studies demonstrated that CLAC significantly improved compliance with achievement of oxygen saturation targets and the number of manual adjustments to the FiO_2_ compared with routine manual oxygen control [12,33]. These effects were present across different SpO_2_ target ranges [12,33]. Subgroup analyses in infants receiving invasive and non-invasive respiratory support showed comparable treatment effects of CLAC and reduced time spent in hypoxia (SpO_2 _< 80%) or hyperoxia (SpO_2_ > 98%) in the two groups [33]. Those studies, however, were of limited duration (24 h periods of automated and manual oxygen control) and tested different devices operating different algorithms [12,33].

Waitz et al. demonstrated that automated oxygen control improved oxygen saturation targeting (76.3% versus 69.1%, *p* < 0.01) among preterm infants receiving different modes of respiratory support (one ventilated; five on non-invasive positive pressure ventilation; nine on CPAP), but did not significantly alter the stability of cerebral tissue oxygenation over 24 h [20]. In addition, CLAC in very-low-birth-weight (VLBW) infants led to a 10% increase in the time spent within the intended oxygen saturation range (77.8% versus 68.5%, *p* < 0.001) and reduced incidence of prolonged hypoxemic episodes (SpO_2 _< 88%, > 180 s), but oxygen tissue saturation was not significantly affected [23].

An RCT in preterm infants on MV or nasal CPAP did not demonstrate any significant differences in the time spent in the target SpO_2_ range (90–95%) and the time spent in hyperoxia (SpO_2_ > 97% when FiO_2 _> 0.21) between infants receiving automated or manual oxygen control [34]. CLAC use was associated with a significant reduction in the time spent in hypoxemia (SpO_2_ < 80%; 0.1% versus 0.6%, *p* = 0.03) and the number of prolonged hypoxemic episodes (0.3 versus 2 per day, *p* = 0.03). The intervention, though, was applied only for 40% of the duration of respiratory support, as infants exited the study if they received support from another device. Therefore, the study results may not be generalisable to other controllers or for the whole duration of oxygen treatment [34]. In a larger RCT that enrolled infants with a mean gestation of 28.8 weeks on day one of life, CLAC increased the total time spent in normoxemia (*p* < 0.001), reduced the frequency of hypoxic and hyperoxic episodes and the number of manual FiO_2_ adjustments, but the monitoring duration was less than 24 h [35].

A recent matched cohort study demonstrated a time-varying effect of CLAC on oxygen saturation targeting [36]. Among 25 extremely preterm infants on different types of respiratory support, there were no significant differences in the achievement of oxygen saturation targets over a seven-week period. Automated oxygen control, however, was associated with a significant increase in the percentage of time spent within SpO_2_ target range during the first two weeks of life for those receiving supplemental oxygen (an increase of 9.9%, *p* = 0.01 in week one and 9.5%, *p* = 0.02 in week two), along with a reduced percentage of time spent in hyperoxia (SpO_2_ > 95%; −9.9% (95% CI [−19.2, −0.2]; *p* = 0.04)).

## 4. Discussion

This review highlights that automated oxygen control improves achievement of oxygen saturation targets in preterm infants receiving non-invasive respiratory support. A systematic review and meta-analysis included five studies in a subgroup of preterm infants on non-invasive ventilation (n = 169) and demonstrated a 15% increase in the time spent in the target SpO_2_ range with automated oxygen control [11]. In addition, a recent Cochrane review and meta-analysis showed that CLAC reduced the percentage of time spent below SpO_2_ target range (MD −5.95% (95% CI −7.98% to −3.92; five studies, 153 infants)) and in hyperoxia (MD −2.31% (95% CI −3.89% to −0.72; five studies, 153 infants; *p* < 0.001)) in infants receiving non-invasive respiratory support but the evidence was of moderate certainty [37]. Zhang et al. included in their meta-analysis studies in infants receiving MV or non-invasive respiratory support, but they did not report subgroup analysis based on the mode of ventilation [38]. Our narrative review focuses on the effectiveness of CLAC in preterm infants receiving different modes of non-invasive respiratory support. In addition, we include studies with prolonged use of the intervention [29,36], which suggest that the effect of CLAC on oxygen saturation targeting may differ over time, with potentially important implications for their use in the early neonatal period. Among extremely preterm non-ventilated infants, CLAC was associated with increased time spent below the target SpO_2_ range in the first few days of life compared with manual oxygen control [29]. In another study, automated oxygen control led to a significant improvement in the achievement of oxygen saturation targets in the first two weeks of life compared with manual control, but the difference was not significant for the total study duration [36].

There are, however, some limitations regarding the included studies. Most of them had a randomised crossover design, a few were prematurely stopped before reaching their predefined sample size [12,29,36,39] and study periods were less than or equal to 24 h [12,17,20,22,23,24,27,30,31,33,35,39]. Therefore, there was limited data available for analysis. Further, as infants were studied at different timepoints, the results may not reflect the effectiveness of CLAC for the whole duration of respiratory support. Indeed, two studies showed a time-varying effect of the intervention [29,36]. Schouten et al. found an increase in the time spent below the target SpO_2_ range with CLAC in the first few days of life that gradually declined [29], but the study was stopped prematurely. Dijkman and co-workers’ results supported the clinical importance of the use of CLAC in the early neonatal period to avoid extremes of oxygen saturations, but their study was limited by the risk of selection bias due to the matched cohort design and the exclusion of non-surviving participants [36]. Furthermore, many of the studies included infants receiving different modes of non-invasive respiratory support or mechanical ventilation and did not report results of subgroups. There were no studies including term-born infants, although some may require non-invasive respiratory support at birth, and previous studies demonstrated that use of CLAC in that population may have similar benefits as in the preterm population [40,41,42]. Furthermore, no data were found on any clinical outcomes for infants on non-invasive respiratory support who were receiving CLAC.

These limitations highlight the need for an adequately powered RCT of CLAC versus manual oxygen control in preterm non-ventilated infants and for the whole duration of non-invasive respiratory support. The trial should aim to determine the effectiveness of automated oxygen control with different modes of non-invasive respiratory support. In addition, staff training along with the use of CLAC can help reduce the incidence of hypoxia and hyperoxia and the risks related to them.

In conclusion, automated oxygen control improves achievement of oxygen saturation targets in preterm infants receiving non-invasive respiratory support by reducing the time spent outside the target SpO_2_ range. Future research should determine the effectiveness of the prolonged use of CLAC with different modes of non-invasive ventilation, with less heterogeneity of the studies in terms of timepoints and monitoring durations, and explore whether it improves long-term outcomes related to oxygen toxicity.

## Figures and Tables

**Table 1:** Included studies.

Author (Year)	Type of Study	Population	Respiratory Support	Sample Size	Algorithm/Controller	Results
Urschitz (2004) [17]	Randomised crossover	<34 weeks GA	CPAP and FiO_2_ > 0.21	12	Rule-based, non-fuzzy (CLAC; Leoni plus; Lowenstein Medical GmbH; Bad Ems, Germany)	Increased time spent in target SpO_2_ range (87–96%) (median (range): 90.5 (59–99.4)% vs. 81.7 (39–99.8)%, *p* = 0.01). Reduced manual FiO_2_ adjustments. Reduced frequency and duration of hyperoxic episodes.
Plottier (2017) [22]	Randomised crossover	<37 weeks GA and if ≤ 4 months, with oxygen requirement	CPAP/nasal HF	20	VDL 1.0 Proportional integral derivative	Increase in time spent within SpO_2_ target range (91–95%) (median (range): 81 (76–90)% vs. 56 (48–63)%, *p* < 0.001). Reduced prolonged hypoxemic and hyperoxemic episodes. Reduced manual FiO_2_ adjustments.
Schwarz (2020) [27]	Two-centre randomised crossover	≤34 weeks GA and frequent hypoxemic episodes	CPAP/MV	19	Rule-based, non-fuzzy Revised and faster algorithm (CLAC; Leoni plus; Lowenstein Medical GmbH; Bad Ems, Germany)	Increased time spent in target SpO_2_ range (90–95%) (mean (SD): 68 (11)% CLAC_fast_ vs. 65 (11)% CLAC_slow_ vs. 58 (11) manual control, *p* < 0.001). Non-inferiority of fast versus slow algorithm.
Dargaville (2022) [28]	Randomised crossover study	<32 weeks gestation and if ≤4 months, with FiO_2_ > 0.21 or hypoxic/apnoeic episodes	Bubble CPAP/HFNC	35	VDL 1.1 Adaptive proportional integral derivative	Increased time spent in target SpO_2_ range (90–94%) (median (range): 81 (72–85)% vs. 58 (51–64)%, *p* < 0.001). Reduced prolonged hypoxemic and hyperoxemic episodes. Reduced manual FiO_2_ adjustments.
Schouten (2024) [29]	Randomised controlled	<28 weeks GA and frequent desaturations and/or FiO_2_ > 0.25	CPAP/NIPPV	23	Rule-based, adaptive CLiO_2_; AVEA ventilator (Vyaire Medical, Mettawa, IL, USA)	Increased time spent in target SpO_2_ range (88–94% and 90–95%) (median (range): 68.7 (59.6–78.6)% vs. 48 (43–56.5)% *p* < 0.001). Reduced time spent in hyperoxia. Varied effect on hypoxia over time.
Zapata (2014) [30]	Randomised controlled	<30 weeks GA and <1000 g	High- or low-flow nasal cannula oxygen	20	Rule-based, fuzzy Automixer (Centro Medico, Imbanaco, Cali, Colombia)	Improved time spent in target SpO_2_ range (85–93%) (mean (SD): 5 (4)% vs. 33.7 (4.7)%, *p* < 0.01). Reduced time spent in hyperoxia. Reduced manual FiO_2_ adjustments. Increased time with SpO_2_: 80–85%.
Reynolds (2019) [24]	Randomised crossover	<37 weeks GA and FiO_2_ ≥ 0.25 with frequent adjustments	HFNC	30	Adaptive (IntellO_2_, Vapotherm precision flow)	Increase in time spent in target SpO_2_ range (90–95%) (median (IQR): 80 (70–87)% vs. 49 (40–57)%, *p* < 0.001). Reduced incidence and duration of prolonged hypoxemic episodes. Increased frequency and reduced duration of hyperoxemic episodes.
Dijkman (2021) [31]	Randomised crossover	<30 weeks GA and FIO_2_ > 0.25	HFNC with Optiflow interface	27	Rule-based algorithm PRICO; Fabian ventilator (Vyaire Medical, Mettawa, IL, USA)	Improved time spent in target SpO_2_ range (88–95%) (mean: 79.5% (95% CI: 76.6–82.5) vs. 68.8% (65.8–71.7), *p* < 0.001). Reduced time spent above and below target range and in severe hypoxia (SpO_2_ < 80%). Higher mean FiO_2_.
Nair (2023) [32]	Randomised controlled	<33 weeks GA	HFNC	60	Adaptive (Oxygen Assist Module, Vapotherm precision flow)	Reduced time in extremes of SpO_2_ (<80% and >98%). Increased time in target SpO_2_ range (90–95%) (median (range): 81 (74–93)% vs. 55 (48–72)%, *p *< 0.001).
Hallenberger (2014) [12]	Multi-centre randomised crossover	<37 weeks GA and FiO_2_ > 0.25	Nasal CPAP/MV	34	Rule-based, non-fuzzy CLAC; Leoni plus (Lowenstein Medical GmbH; Bad Ems, Germany)	Increase in time spent in target SpO_2_ range (centre 1: 90–95%, centre 2: 80–92%, centre 3: 83–93%, centre 4: 85–94%) (median (range): 71.2 (44–95.4)% s 61.4 (31.5–99.5)%, *p* < 0.001). Reduced manual FiO_2_ adjustments.
Van Kaam (2015) [33]	Randomised crossover	<33 weeks GA	MV/non-invasive respiratory support	80	Adaptive A-FiO_2_; AVEA ventilator (Vyaire Medical, Mettawa, IL, USA)	Increased time spent in target SpO_2_ range (89–93% or 91–95%) (mean (SD): 62 (17)% vs. 54 (16)%, *p* < 0.001 in lower range and 62 (17)% vs. 58 (15)%, *p* < 0.001 in higher range). Reduced time spent in hypoxemia and hyperoxemia.
Waitz (2015) [20]	Randomised crossover	<30 weeks GA and frequent hypoxemic episodes	MV/CPAP/NIPPV	15	Rule-based, adaptive CLiO_2_; AVEA ventilator (Vyaire Medical, Mettawa, IL, USA)	Increased time in target SpO_2_ range (88–96%) (mean (SD): 76.3 (9.2)% vs. 69.1 (8.2)%, *p* < 0.01). Reduced incidence of prolonged hypoxemic episodes. No effect on cerebral tissue oxygenation.
Gajdos (2019) [23]	Randomised crossover	<30 weeks GA and frequent hypoxemic episodes	MV/CPAP/NIPPV	12	Proportional integral derivative SPO_2_C; Sophie infant ventilator (Fritz Stephan Gackenbach, Germany)	Increased time in target SpO_2_ range (88–96%) (mean (SD): 77.8 (7.1)% vs. 68.5 (7.7)%, *p* < 0.001). No effect on oxygen tissue saturation.
Nair (2023) [34]	Randomised controlled	<33 weeks GA, within 72 h of life	Nasal CPAP/MV	44	Rule-based, adaptive CLiO_2_; AVEA ventilator (Vyaire Medical, Mettawa, IL, USA)	Reduced time spent in hypoxemia (SpO_2_ < 80%) (median (IQR): 0.1 (0.07–0.7)% vs. 0.6 (0.2–2)%, *p* = 0.03). Reduced incidence of prolonged hypoxemic episodes.
Rocha (2025) [35]	Randomised controlled	<33 weeks GA, within 24 h of life	Nasal CPAP/MV	89	Rule-based PRICO; Fabian ventilator (Acutronic, Hirzel, Switzerland)	Increased time spent in target SpO_2_ range (90–94%) β = 81.5; 95%CI: 47.9–115.2, *p* < 0.001. Reduced number of manual adjustments to the FiO_2_. Reduced frequency of hypoxemic and hyperoxemic episodes.
Dijkman (2025) [36]	Matched cohort	<28 weeks GA	CPAP, MV, HFOV	25	Rule-based PRICO; Fabian ventilator; Vyaire Medical, Mettawa, IL, USA	Increased time spent in target SpO_2_ range during the first two weeks of life (88–95%). 1st week: mean difference: 9.9 (95% CI: 3.1–16.7)%, *p* = 0.01) 2nd week: mean difference: 9.5 (95% CI: 1.4, 17.6)%; *p* = 0.02

## Data Availability

Not applicable.

## Abbreviations

The following abbreviations are used in this manuscript:

BPD	Bronchopulmonary dysplasia
CLAC	Closed-loop automated oxygen control
CPAP	Continuous positive airway pressure
FiO_2_	Fraction of inspired oxygen
HFNC	High-flow nasal cannula
MV	Mechanical ventilation
RCT	Randomised controlled trial
RDS	Respiratory distress syndrome
ROP	Retinopathy of prematurity
SpO_2_	Peripheral oxygen saturation
VLBW	Very low birth weight
